# Trends in Choosing Place of Delivery and Assistance during Delivery in Nanded District, Maharashtra, India

**DOI:** 10.3329/jhpn.v29i1.7568

**Published:** 2011-02

**Authors:** Geeta S. Pardeshi, Shashank S. Dalvi, Chandrakant R. Pergulwar, Rahul N. Gite, Sudhir D. Wanje

**Affiliations:** ^1^ Department of Preventive and Social Medicine, Dr. Shankarrao Chavan Government Medical College, Nanded 431 605, India; ^2^ District Health Office, Zilla Parishad, Nanded 431 605, India

**Keywords:** Community-based studies, Cross-sectional studies, Descriptive studies, Delivery, Rural health services, India

## Abstract

Delivery in a medical institution promotes child survival and reduces the risk of maternal mortality. Manyinitiatives under the National Rural Health Mission (NRHM) focus on increasing the institutional deliveries. This study describes the trends in choosing place of delivery in Nanded district at the end of the first phase of the mission. Key informants were interviewed to document the initiatives under NRHM implemented in the district. A cross-sectional descriptive study was conducted in 30 villages selected using one stage cluster-sampling method. A house-to-house survey was conducted in June 2009. A set of structured open-ended questionnaire was used for interviewing all women who had delivered during January 2004–May 2009. The outcomes studied were place of delivery and assistance during delivery. Analysis was done by calculating chi-square test and odds ratio. Interventions to improve the quality of health services and healthcare-seeking behaviour were implemented successfully in the district. The proportion of institutional deliveries increased from 42% in 2004 to 69% in 2009. A significant increase was observed in the proportion of institutional deliveries [60% vs 45%; χ^2^=173.85, p<0.05, odds ratio (OR)=1.8 (95% confidence interval (CI) 1.65-1.97)] in the NRHM period compared to the pre-NRHM period. The deliveries in government institutions and in private institutions also showed a significant rise. The proportion of deliveries assisted by health personnel increased significantly during the NRHM period [62% vs 49%; χ^2^=149.39; p<0.05, OR=1.73, 95% CI 1.58-1.89] However, less than 10% of the deliveries in the home (range 2-9%) were assisted by health personnel throughout the study period. There was a wide geographic variation in place of delivery among the study villages. The results showed a significant increase in the proportion of institutional deliveries and deliveries assisted by health personnel in the NRHM period. Since a less proportion of deliveries in the home is conducted by health personnel, the focus should be on increasing the institutional deliveries. Special and innovative interventions should be implemented in the villages with a less proportion of institutional deliveries.

## INTRODUCTION

The place of delivery is a crucial factor which affects the health and well-being of the mother and the newborn (1). Institutional deliveries provide easy access to skilled assistance, drugs, equipment, andreferral transport. One of the sociodemographic goals mentioned in the National Population Policy 2000 of India is to achieve 80% institutional deliveries and 100% deliveries to be assisted by skilled health personnel by 2015 (2). These two interventions have also been identified as important initiatives to reduce the maternal mortality ratio—the fifth Millennium Development Goal (3).

The National Family Health Survey (NFHS)-3 (2005-2006) reported that 31% of deliveries in rural India and 51% of deliveries in rural Maharashtra took place in an institution in the three-year period before the survey was conducted (4, 5). The NFHS-3 also reported that health personnel assisted 40% of deliveries in rural India and 57% of deliveries in rural Maharashtra in the three-year period before the survey (4, 5). The National Rural Health Mission (NRHM), being implemented in the country from 2005 to 2012 (6), focuses on expanding and strengthening the existing rural health services. Simultaneously, it also includes various initiatives to promote institutional deliveries. The NRHM is being implemented in Nanded district, Maharashtra state in western India since April 2006.

This study describes the trends in institutional deliveries and assistance during delivery in the pre-NRHM and the NRHM period. We also document the various initiatives taken under the NRHM to increase the proportion of institutional deliveries in Nanded district.

## MATERIALS AND METHODS

A community-based cross-sectional study was conducted in 30 villages selected using the single-stage cluster-sampling method. The village-wise list ofthe 2001 census provided the sampling frame for selecting the villages.

Key informants, i.e. District Health Officer, Assistant District Health Officer, a few selected medical officers, and auxiliary nurse-midwives (ANMs), were interviewed to document the various initiatives taken under NRHM to increase the proportion of institutional deliveries in Nanded district.

The study included all women who were permanent residents of the sample villages, who had delivered during 1 January 2004–31 May 2009. Data were collected by a house-to-house survey in June 2009. A pre-tested open-ended questionnaire was used for collecting data. The outcomes studied were place of delivery and assistance during delivery. If the women were not present in the house at the time of the survey, proxy information was obtained from family members. Such proxy information was collected in the case of 53 deliveries.

Institutional delivery was defined as delivery in either government institutions (subcentres, primary health centres, first referral units, and district hospitals) or private clinics. Assistance by health personnel was defined as assistance by a doctor, a nurse, or an ANM.

The difference in the proportion of institutional and non-institutional deliveries and assistance by health professionals and others was assessed using the chi-square test and calculating the odds ratio with 95% confidence interval. Ethical clearance for conducting the study was obtained from the Ethical Committee, Dr. Shankarrao Chavan Government Medical College, Nanded.

## RESULTS

Many initiatives under NRHM are aimed at improving the proportion of institutional deliveries. The interventions have been implemented phase-wise since April 2006. These include interventions to improve the quality of services through staff appointments, development of infrastructure, provision of equipment, training and capacity-building, and public-private partnership. Provision of funds and strict monitoring ensured the successful implementation of the interventions. Attempts were made to improve the use of healthcare services through cash incentives, information, education, and communication (IEC) activities, and appointment of Rugna Kalyan Samitees (Table 1).

**Table 1.** Interventions under NRHM aimed at improving the proportion of institutional deliveries

Intervention	Details
Cash incentives	Under the Janani Suraksha Yojana (JSY), cash incentives are provided to mothers to get them to deliver their babies in a health facility. It covers all pregnant women belonging to households of below poverty-line (BPL) category, scheduled caste, or scheduled tribes, over 19 years, and up to two livebirths
Staff appointments	Staff vacancies were identified as a major hurdle in the implementation of the programme. Hence, new appointments were made under which 149 of the 151 posts of medical officers in the district were filled, and 91 of the 101 posts of medical officers and specialists at the first referral units (FRUs) were filled. A new cadre of general nurse-midwives (GNMs) was appointed at the primary health centres (PHCs)
Public-private partnership	This was attempted through the mother NGO scheme, accreditation of private clinics, and appointment of specialists on a contract basis
Provision of equipment	Blood-storage facilities were provided at three first referral units, and baby-warmers, inverters, oxygen cylinders, and solar heaters were provided at the PHCs
Development of infrastructure	Delivery-rooms were constructed in 161 of the 374 subcentres, and repairs and renovations were made in 26 of the 63 PHCs in the first phase of the programme
Training and capacity-building	Three medical officers were trained for lifesaving anesthesia skills and posted at the FRUs, and medical officers and paramedical staff were trained in essential obstetric care
Role of Rugna Kalyan Samitees	Rugna Kalyan Samitees are the registered societies involving people's representatives in the management of the hospital. In Nanded district, they have provided funds for provision of transport facilities during emergency, providing food to patients and escorts during inpatient stay after delivery, presenting clothes and coconut to mother at the time of discharge, which is a traditional way of honouring guests at the time of departure. These activities have helped improve the image of the public institutions
IEC activities	A group of 45 health workers and anganwadi workers were selected and trained to conduct IEC on the topic of maternal and child healthcare. Messages on the importance of institutional deliveries were broadcast on the radio regularly
Monitoring	The status of institutional deliveries was discussed on a priority basis in the monthly meetings at the District Health Office. Pregcare, a software developed by the Assistant District Health Officer at Nanded, was used for tracking all antenatal cases and monitoring their antenatal care, intranatal care, and postnatal care. All the subcentres were linked through the PHCs to taluka medical officer and finally to the district headquarters
Availability of funds	Funds were available from NRHM and the Human Development Mission which supported the institutional deliveries as it contributed to reduce infant mortality, an important component of human development index

IEC=Information, education, and communication; NGO=Non-government organization; NRHM=Natio-nal Rural Health Mission

### Trends in choosing place of delivery andassistance during delivery

**Fig. 1.**
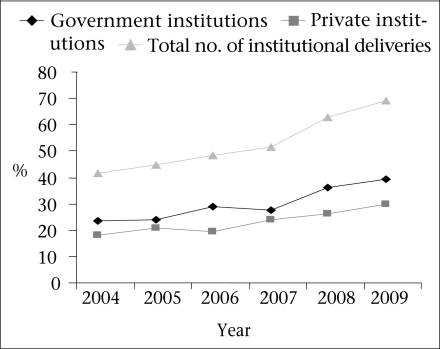
Trends in choosing places for institutional deliveries, 2004–2009

A rising trend was observed after 2007 in the proportion of institutional deliveries, the proportion of deliveries in government institutions, and the proportion of deliveries in private institutions (Fig. 1).

The proportion of institutional deliveries increased from 42% in 2004 to 69% in 2009. The proportion of deliveries in the government institutions increased from 24% to 39%, and the proportion of deliveries in the private institutions increased from 18% in 2004 to 30% in 2009. The proportion of deliveries assisted by health personnel increased from 50% in 2004 to 70% in 2009 (Table 2).

**Table 2.** Trends in choosing places and seeking assistance in institutional deliveries, 2004-2009

		*Institutional deliveries*						*Assistance by health-personnel during delivery*	
Year	Total no. of deliveries	Government institutions		Institutional deliveries		Total			
		No.	%	No.	%	No.	%	No.	%
2004	1,530	361	23.59	636	41.57	361	23.59	703	45.95
2005	1,419	341	24.03	638	44.96	341	24.03	695	48.98
2006	1,576	455	28.87	759	48.16	455	28.87	796	50.51
2007	1,477	405	27.42	759	51.39	405	27.42	801	54.23
2008	1,418	514	36.25	888	62.62	514	36.25	929	65.51
2009	793	313	39.47	550	69.36	313	39.47	557	70.24

A significant increase was observed in the proportion of institutional deliveries and deliveries in the government institutions and in the private institutions in the NRHM period compared to the pre-NRHM period (Table 3). Women were 1.8 times more likely to deliver in an institution in the NRHM period compared to the pre-NRHM period. Women were 1.46 times more likely to deliver in a government institution and 1.47 times more likely to deliver in a private institution in the NRHM period compared to the pre-NRHM period (Table 3). There was a significant increase in the proportion of deliveries assisted by the health personnel, with 1.73 times more chances of such assistance in the NRHM period.

**Table 3.** Comparison of proportion of place of deliveries and assistance during deliveries

Delivery characteristics	NRHM (n=3, 688)		Pre-NRHM (n=4, 525)		χ^2^	OR (95% CI)
	No.	%	No.	%		
Institutional	2,197	59.57	2,033	44.93		
Non-institutional	1,491	40.43	2,492	55.07	173.85	1.8 (1.65-1.97)
Delivery in government institutions	1,232	33.41	1,157	25.57		
Others	2,456	66.59	3,368	74.43	60.11	1.46 (1.32-1.60)
Delivery in private institutions	965	26.17	876	19.36		
Others	2,723	73.83	3,649	80.64	53.74	1.47 (1.33-1.63)
Assistance by health personnel	2,287	62.01	2,194	48.49		
Assistance by relatives and neighbours	1,401	37.99	2,331	51.51	149.39	1.73 (1.58-1.89)

CI=Confidence interval; NRHM=National Rural Health Mission; OR=Odds ratio

Throughout the study period, less than 10% of the total number of deliveries in the home were assisted by the health personnel (Table 4).

**Table 4.** Proportion of home-deliveries conducted by health personnel

Year	Total no. of home-deliveries	Home-deliveries assisted by health personnel	
		No.	%
2004	894	67	7.50
2005	781	71	9.09
2006	817	37	4.52
2007	718	42	5.84
2008	530	41	7.73
2009	243	7	2.88

The places of delivery during June 2008–May 2009 in the 30 villages were compared (Table 5). A wide variation was observed in the place of delivery among the study villages. In six villages, more than 50% of the deliveries had taken place in the government institutions. In three villages, no deliveries had occurred in the private institutions while, in four villages, more than 50% of the deliveries occurred in the home.

**Table 5.** Distribution of place of delivery in the study villages

% of total deliveries	Place of delivery					
	Government institution (n=30)		Private institution (n=30)		Home (n=30)	
	No.	%	No.	%	No.	%
<5	0	0	3	10.00	0	0.00
5-25	5	16.67	16	53.33	4	13.33
26-50	19	63.33	11	36.67	22	73.34
51-75	4	13.33	0	0.00	4	13.33
>75	2	6.67	0	0.00	0	0.00

## DISCUSSION

An increasing trend was observed in the proportion of institutional deliveries and deliveries assisted by health personnel over the study period which has been accelerated from 2007 as a result of the interventions under NRHM.

NRHM is being implemented in a campaign mode in which many initiatives, such as development of infrastructure and appointment of staff, are implemented phase-wise (7). The healthcare-seeking behaviour for intranatal care is a complex and multi-factorial entity. A number of social, economic, cultural and geographic factors are known to be related to the choice of the place of delivery (8-11). Hence, a multi-pronged and holistic approach, which addresses these issues, is needed to improve the number of institutional deliveries. The initiatives under NRHM address many of these issues, such as economic aspects, improving the quality of health services, behaviour change, etc. Many initiatives, such as Janani Suraksha Yojana, appointment of Rugna Kalyan Samitees, and development of infrastructure, are included in the basic framework of NRHM and have been implemented throughout the country under this mission. An impressive range of innovative approaches has also been adopted to address the local needs and gaps while implementing the programmes of NRHM (12). The roles of the Rugna Kalyan Samitees and Pregcare have been the innovative activities carried out in Nanded district.

Nearly 69% of the total number of deliveries (n=2, 211) in 2008-2009 in rural Nanded were conducted in ins-titutions. A substantial increase in the proportion of institutional deliveries has been reported since the implementation of NRHM (13, 14).

In this study, 39% of the total number (n=2, 211) of deliveries in 2008-2009 were conducted in the government institutions and 30% in the private institutions. As the existing gaps in healthcare provision are met, it is expected that the contribution of public institutions in promoting institutional deliveries will substantially increase. The proportion of deliveries in the private institutions is also on the rise. However, studies have reported that the cost of delivery in the private sector is many times higher than that in the public sector (15, 16). Public-private partnership and development of collective payment schemes to meet delivery-care needs have been recommended (16).

By May 2009, 70% of the deliveries in rural Nanded were assisted by the health personnel. All deliveries should be assisted by the health personnel if the sociodemographic goal is to be met. In this study, very few deliveries in the home were assisted by the health personnel. In a study of the national trends, two different trends in the proportion of deliveries attended by health personnel have been reported in six countries studied (17). One reflects a delivery-care model in which virtually all births are attended by health personnel in health facilities with a shift away from professional deliveries in the home towards professional deliveries in a health facility. In another model, an increase in attendance of professionals at delivery is driven by an increase in the number of births in the home by health personnel. The present study indicates that, in the first phase of NRHM, an increase in the proportion of institutional deliveries has contributed to an increase in the assistance during delivery by health personnel. The skilled attendance can only be provided when health professionals operate within a well-functioning health system, i.e. an enabling environment where drugs, equipment, supplies, and transport are available. However, increasing the proportion of institutional deliveries would have profound resource and logistical implications, and the health system needs to be ready to cater to the increasing demand.

Overall, there has been an increase in the proportion of institutional deliveries, yet regional variation remains within the country and between the districts too (18). The analysis of the institution-wise database on delivery status over a two-year period after the implementation of NRHM in Orissa showed that there was an overall 33% increase in institutional deliveries in the state but with a wide variation within the districts (19). In our study, there was a wide variation in the place of delivery among the villages which is the beginning of the regional variation. It is necessary to focus on the villages which report a high proportion of non-ins-titutional deliveries.

This is a population-based study with a large sample-size which ensures that the findings are representative of the experiences of rural women of Nanded district. However, one of the limitations of the study is the proxy information in the case of 53 (0.64%) deliveries obtained during the survey. The increasing trend with time would lead to underestimation of the proportion of institutional deliveries for 2009. It is, therefore important to note that, although 2009 is mentioned in the study, the exact period of the study was only the first six months of 2009.

**Conclusions**

A significant increase was observed in the proportion of institutional deliveries and deliveries assisted by health personnel since the implementation of NRHM. It is necessary to sustain the achievements and further increase the coverage of the initiatives under NRHM. The villages with low proportion of institutional deliveries should be provided with specific interventions focusing on their needs and innovative approaches.

## ACKNOWLEDGEMENTS

The authors acknowledge the contribution of all the Medical Officers, Health Workers, and Health Assistants who contributed in data collection. They also acknowledge the cooperation of the villagers who participated in this study.
